# Prevalence of Early Postnatal-Care Service Utilization and Its Associated Factors among Mothers in Hawassa Zuria District, Sidama Regional State, Ethiopia: A Cross-Sectional Study

**DOI:** 10.1155/2021/5596110

**Published:** 2021-05-13

**Authors:** Shambel Yoseph, Azmach Dache, Aregahegn Dona

**Affiliations:** ^1^Hawassa Zuria District Health Office, Hawassa Zuria, Sidama Regional State, Ethiopia; ^2^Department of Social and Population Health, Yirgalem Hospital Medical College, Yirgalem, Sidama, Ethiopia

## Abstract

**Background:**

A postnatal care given after childbirth is a critical care to promote health and to prevent complications of the mother and newborn. However, utilization of this service is low in Ethiopia, and little is known about its coverage and determinants. Thus, this study aimed to assess the prevalence of early postnatal-care service utilization and its associated factors among mothers in Hawassa Zuria district, Sidama Regional State, Ethiopia.

**Methods:**

A cross-sectional study was conducted from 20 February to 20 March 2020 in Hawassa Zuria District among randomly selected 320 mothers. Data were collected by using interviewer-administered structured questionnaires. Data entered were into Epi data version 3.1 and exported to SPSS version 26 for analysis. Descriptive, bivariable, and multivariable logistic regression analysis with odds ratio and 95% confidence interval were conducted. A *P* value <0.05 was considered a statistically significant association. Finally, the results were presented by texts, tables, and figures.

**Result:**

The prevalence of early postnatal-care service utilization was 29.7% (95% CI = 24.7, 35.5). Age below 25 years [AOR = 3.2 (95% CI = 1.37, 7.48)], having planned and supported pregnancy for last birth [AOR = 2.2 (95% CI = 1.13, 4.38)], having information about obstetric danger signs [AOR = 2.1 (95% CI = 1.25, 3.78)], and having positive attitude on use postnatal services [AOR = 3.5 (95% CI = 1.94, 6.32)] were factors associated with early postnatal-care utilization.

**Conclusion:**

The finding revealed that early postnatal-care utilization in the study area was low. Strengthening family planning services, giving information on obstetrics danger signs, and creating awareness about postnatal care will improve uptake of the service in a timely manner.

## 1. Background

Early postnatal care (EPNC) is the care given to the mother and her newborn immediately after birth and up to the first seven days of life that marks the establishment of a new phase of family life for women and their partners and the beginning of the lifelong health record for newborn [1]. According to the World Health Organization (WHO) recommendation, the mothers have at least three postnatal-care (PNC) follow-ups by the health professionals or trained community health workers within the first 24 hours, 2-3 days, and at 7 days after delivery [2].

The care of a woman and her baby in the immediate hours, days, and weeks following birth can make an enormous difference to their long-term health and well-being. It leads to a dramatic fall in the maternal mortality rate and helps to establish and maintain contact with a number of health services needed in the short and long terms [3].

Even though it is the most neglected period for the provision of quality care services, the first week after delivery is a critical phase in the lives of a mother and her newborn. Lack of appropriate care during this period could result in a significant ill-health and even death [4].

Globally, the majority of maternal and infant mortality occurs in the first month after birth. Almost half of the postnatal maternal deaths occur within the first 24 hours, 66% occur during the first week after delivery, and one million newborns die on the first day of life. The main reasons for these easily preventable problems were poor quality of services, weak community-based heath practice, gender inequality, and poor women-centered maternity care [5, 6].

Although considerable progress has been made globally to improve maternal health, low utilization of EPNC is one of the major causes of maternal mortality and morbidity in the developing countries. Two regions, sub-Saharan Africa and South-East Asia, account for 86% of maternal mortality worldwide, and most of that occurred within 7 days after delivery. Additionally, the majority of women are not getting a PNC visit within 2 days of childbirth due to cultural practices, lack of education, shortage of income, and lack of awareness on the availability of the services [7]. In most developing countries, PNC may only be accessed if provided through home visits because of the geographical, financial, cultural, and social barriers that limit care outside the home during the early postnatal period [8].

The government of Ethiopia endorsed a strategy which is aimed at strengthening the health system to provide quality care. The Health Sector Transformation Plan 2015/16 set a target of up to 95% postnatal coverage by the year 2020 to transform all health districts by creating a high performance of primary health-care units and model kebeles [9]. However, the 2016 Ethiopian Min Demographic Health Survey reported that four in five women (81%) did not receive any PNC checkup [5], and in 2019, only 34% of women received PNC check-up in the first 2 days after birth [10].

Even though the Ethiopian government is providing free maternal and child PNC services regardless of the socioeconomic status of the women to ensure access to the community-based facilities, the maternal mortality ratio remains high at the national level, and low utilization of EPNC continued as the leading cause of maternal morbidity and mortality [11].

Despite the fact that it has a very significant and positive impact on the reduction of maternal and newborn morbidity and mortality, EPNC service is yet neglected and less attention has been given to it. Therefore, the aim of this study was to assess the prevalence of EPNC service utilization and its associated factors among mothers in Hawassa Zuria district, Sidama Regional State, Ethiopia, in 2020.

## 2. Methods and Materials

### 2.1. Study Setting and Period

The study was conducted in Hawassa Zuria district. Hawassa Zuria District is one of 36 districts in Sidama Regional State, Ethiopia, which is located 21 kilometers away from Hawassa, the capital of the region. The district has an estimated population number of 168,188 according to the current population projection and the estimated deliveries were 5,819. The district has 23 kebeles including three town kebeles. Currently, there are 4 public health centers, 1 primary hospital, 23 health posts, and 5 primary private clinics. The study was conducted from 20 February to 20 March 2020.

### 2.2. Study Design and Population

A community-based cross-sectional study was conducted among postnatal mothers who had been living in Hawassa Zuria district. All mothers who gave birth six months prior to this survey in the selected kebeles were included in this study. Those who were critically sick and unable to give a response during the data collection period were excluded.

### 2.3. Sample Size Determination and Sampling Procedures

The sample size was determined by using the single population proportion formula with the following assumptions: considering 25.3% of postnatal-care service utilization within seven days taken from the previous study [12], 95% confidence interval, 5% marginal of error, and 10% nonresponse rate, the final sample size calculated was 320 participants. From the 23 kebeles found in Hawassa Zuria district, six kebeles were selected by a simple random sampling method. After the proportional allocation of a sample size to each kebele based on its study participants, a simple random sampling technique was used to select 320 study participants. Delivery registration book and family folders in the selected kebeles were served as a sampling frame to select the eligible women to be enrolled in this study.

### 2.4. Data Collection Tools, Procedures, and Quality Assurance

Data were collected by using interviewer-administered structured and pretested questionnaires developed by reviewing the related literature. The questionnaire contains sociodemographic and economic factors, reproductive and obstetrics factors, awareness of the mother on postnatal-care service, the attitude of mothers towards postnatal-care services as well as health-care providers, and facility-related factors. Data were collected by six nurses with diploma qualifications. One nurse with Bachelor of Science qualification was assigned as a supervisor. To control the quality of the data, a properly designed data collection tool was developed in English and translated into the local language (Sidaamu Afoo) and back to English by language experts to check its consistency. All data collectors and supervisors were trained for one day by the principal investigator before starting the actual data collection. Training was given on the general objective of the study, contents of the tool, and how to approach the study participants. Before starting the actual data collection, the tool was pretested on 5% of the sample population at one kebele outside the study area and necessary measures were taken accordingly. Collected data were checked for its completeness and consistency before starting actual data entry. The dependent variable was early postnatal-care service utilization that was measured by a ‘yes' or ‘no' response. Positive (yes) responses were validated by asking about the types of services utilized. Independent variables were sociodemographic and economic factors, reproductive and obstetrics factors, awareness of the mother on postnatal-care service, the attitude of mothers towards postnatal-care services as well as health-care provider, and facility-related factors.

### 2.5. Data Processing and Analysis

After data cleaning and checking its completeness, data were coded and entered into Epi data version 3.1 software and finally exported to statistical software for social science (SPSS) version 21 for analysis. Descriptive analysis was performed for each predictor variable, and cross tabulation was performed to see the distribution of predictor variables in relation to outcome variable. The goodness-of-fit of the model was also checked by Hosmer–Lemeshow goodness-of-model fit. Bivariable analysis was performed for each independent variable with the outcome variable, and variables with a *P* value of 0.20 and below were considered as candidates for multivariable logistic regression analysis to control possible confounders and to get the final model. Adjusted odds ratio (AOR) with 95% confidence interval (CI) was calculated to determine the presence and strength of association among predictors and outcome variable. A *P* value of less than 0.05 was used to consider statistically significant variables. Finally, the results were described by texts, figures, and tables.

### 2.6. Operational Definitions

  Used early postnatal-care services: if the mother used at least one PNC service in the first seven postpartum days that was provided by the health professionals regardless of the place of delivery
  Awareness on the postnatal danger signs: those mothers mentioned at least one obstetrics danger sign occurred after delivery
  Awareness of early postnatal care: mothers who had information about at least one postnatal-care service provided within one week after delivery
  Positive attitude on postnatal care: those correctly scored above mean from attitude-related items

## 3. Results

### 3.1. Sociodemographic Characteristics

From 320 participants planned for this survey, all of them were interviewed. The mean age of the respondents was 29.1 ± 5.8 years. Of the interviewed respondents, 257 (80.3%) were protestant. About 263 (82.2%) of them were from the Sidama ethnic group. 311 (97.2%) of them were married, and 151 (47.2%) attended primary schools. 254 (79.4%) of the respondents had either TV and/or radio in their house (Table 1).

### 3.2. Obstetrics Complications and Reproductive Characteristics of the Study Participants

One hundred and ninety-four (60.6%) of the respondents had less than four children. Two hundred and sixty-one (86.1%) had one or more ANC follow-ups during their last pregnancy; out of these, only 96 (36.8%) had four or more ANC visits. The common complications faced after delivery were vaginal bleeding (9.1%) followed by severe abdominal pain (6.6%) (Table 2).

### 3.3. Awareness and Attitude of the Study Participants towards EPNC Services

More than half (55%) had awareness on the EPNC services. Regarding obstetric danger signs, 135 (42.2%) have mentioned at least one danger sign. The most commonly mentioned signs were vaginal bleeding (26.7%) followed by severe abdominal pain (26.7%). Concerning respondents' attitudes toward EPNC utilization, the mean attitude score was 25 (Table 3).

### 3.4. Health Facility and Health Workers Related Factors for EPNC

**Figure 1:**
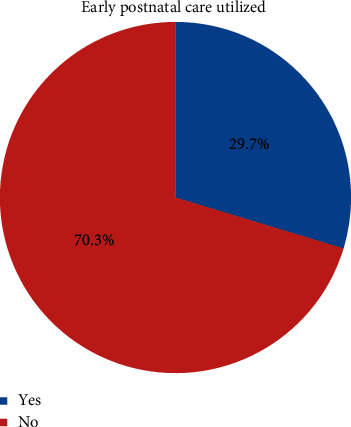
Magnitude of early postnatal-care services utilized by the respondents in Hawassa Zuria District, Sidama Regional State, Ethiopia, 2020.

Concerning the time taken to get to the services, more than half (55.9%) of them traveled at least 30 minutes to reach the health facility. The majority of the respondents (89.1%) used health facility delivery services for their last birth. Only 95 (29.7%) of them used EPNC for recent delivery (Figure 1). The main reasons mentioned by the respondents for not using EPNC services were lack of information followed by lack of time (Table 4).

### 3.5. Factors Associated with EPNC Service Utilization

To identify the association of independent variables with the outcome variable (utilization of EPNC), both bivariate and multivariable logistic regression analysis were performed. In bivariable logistic regression analysis age of the mother, educational status of the mother, the number of children alive, history of ANC follow-up for the last pregnancy, condition of the last pregnancy, complications faced during the last pregnancy and after delivery, awareness on obstetrics danger signs, and attitude towards EPNC services were variables associated with EPNC service utilization.

In multivariable logistic regression analysis after controlling for potential confounder, age of the mother, having planned and supported pregnancy, having awareness on obstetric danger signs, and having a positive attitude towards EPNC services were the factors statistically associated with the outcome variable (EPNC service utilization).

Mothers whose age was below 25 years were 3.2 times more likely to utilize early postnatal-care services when compared with those whose age was above 35 years [AOR = 3.2, 95% CI (1.37, 7.53)]. Those mothers who had planned and supported pregnancy for their last birth were 2.28 times more likely to utilize early postnatal-care services than those who had unplanned and unsupported pregnancy for their last pregnancy [AOR = 2.28, 95% CI (1.15, 4.52)].

Mothers who had awareness on obstetric danger sign and symptoms were 2.2 times more likely to utilize early postnatal-care services when compared with those who had no awareness [AOR = 2.2, 95% CI (1.27, 3.87)]. Mothers who had a positive attitude towards EPNC-care services were 3.5 times more likely to utilize early postnatal care when compared with those who had negative attitude [AOR = 3.5, 95% CI (1.95, 6.33)] (Table 5).

## 4. Discussion

This study evaluated the EPNC practices in Hawassa Zuria district, Sidama Regional State, Ethiopia. According to this study, the level of EPNC service utilization was found to be 29.7% (95% CI: 24.7, 35.5). This result was similar to the findings from the study conducted in rural Myanmar (25.20%), Northern Shoa, Ethiopia (28.4%), and Debre Markos town (33.5%) [13–15]. The reason for this consistency may be the similarity of the study design and setting and sociodemographic characteristics of the study participants.

This result was higher than the previous findings from eastern Uganda (15.4%), Aseko District (23.7%), and Mertule Mariam District (19%) [16–18]. This may be attributed to the time difference, and there could be improvement in accessing and utilizing health-care service through time.

However, this result was lower when compared with previous findings in Benin (68.42%), Addis Ababa (65.6%), and Shebe Sombo district, Jimma Zone (58.5%) [19–21]. The most likely reason for this discrepancy may be due to the place of residence and geographical variation. The difference also may be due to that the previous studies considered postpartum mothers up to forty-two days. As a result, the chance of utilizing the service could be increased when compared with this study which considered only the first week of the postpartum period.

In view of addressing the different factors in influencing the practices of EPNC services in the study area, an attempt was made to examine the associations between various explanatory variables and the outcome variables. The study identified four variables which have positive significant associations with early postnatal-care service utilization.

This study revealed that younger mothers whose age was below 25 years were 3.2 times more likely to practice EPNC services [AOR = 3.2, 95% CI (1.37, 7.53)] when compared with older ones. This was also seen in previous studies conducted in Shebe Sombo and Fiche town, Oromia Region, Ethiopia [21, 22]. This may be because younger mothers were more likely to have greater exposure to mass media and more access to education. However, this finding was in contrast with previous findings from Farta district, south Gondar Zone in Ethiopia and Tanzania [23, 24].

The odds of having EPNC services were 2.28 times more likely [(AOR = 2.2, 95% CI (1.15, 4.52)] among those mothers who had planned and supported pregnancy when compared with those whose pregnancy was unplanned and unsupported. This finding was in line with the previous study conducted in Debre Tabour town and Tigray Region, Ethiopia [25, 26]. This may be because mothers' interests to use EPNC services can be increased based on the condition of the pregnancy, whether they are planned and supported.

Awareness on potential postnatal danger signs has a positive association with EPNC utilization. Mothers who have mentioned at least one obstetric danger sign and symptom were 2.2 times more likely [AOR = 2.2, 95% CI **(**1.27, 3.87)] to utilize EPNC service as compared to those who had no awareness on obstetric danger signs and symptoms This finding was supported by the previous study conducted in Diga district, West Wollega, and Lemo district, Ethiopia [27, 28]. This can be explained by the fact that awareness of obstetrics danger sign is an important factor in triggering and motivating the mothers to seek health-care services as soon as possible with the aim of preventing, diagnosing, and even treating the case if problem exists.

Finally, attitude towards EPNC service also had a positive association with EPNC service utilization. Mothers who had a positive attitude towards EPNC were 3.5 times more likely to utilize EPNC services [AOR = 3.50, 95% CI (1.95, 6.33)] when compared with those who had negative attitude. This finding was consistent with that of previous studies which reported that a respondent's attitude was a critical factor in encouraging a mother to receive EPNC services [29]. This result was also supported by the previous study conducted in Bahi District, Tanzania [30]. The most likely explanation for this might be that a positive attitude is the most valuable precondition for any healthy behavior; as a result, it might encourage adaptation and practices of healthy personal behavior that help to improve an individual's health status.

## 5. Conclusions

This study revealed that the coverage of early postnatal-care service utilization was low in the study area. Age of the respondents, having planned and supported pregnancy, having awareness on obstetrics danger signs, and having a positive attitude towards EPNC service were factors significantly associated with the utilization of EPNC service. Strengthening family planning services, giving information on obstetrics danger signs and symptoms, and creating awareness on benefits of EPNC will increase uptake of the service in a timely manner.

## Figures and Tables

**Table 1:** Sociodemographic characteristics of the study participants in Hawassa Zuria district, Sidama Regional State, Ethiopia, 2020.

Variables	Categories	Frequency	Percentage
Age in years	<25	125	39.1
	25–35	119	37.2
	>35	76	23.8

Religion	Protestant	257	80.3
	Muslim	44	13.8
	Orthodox	19	5.9

Residence	Urban	25	7.8
	Rural	295	92.2

Marital status	Married	290	90.6
	Unmarried	19	5.9
	Widowed	11	3.5

Mother's educational status	Secondary and above	69	21.6
	Primary school	151	47.2
	No formal education	100	31.3

Husband educational status	Secondary and above	139	43.5
	Primary school	162	50.6
	No formal education	19	5.9

Mother's occupation	Employer	27	8.4
	Student	64	20
	Merchant	103	32.2
	Housewife	126	39.4

Husband's occupation	Farmer	131	40.9
	Employer	59	18.4
	Merchant	110	34.4
	Daily labor	20	6.3

Monthly income	>1500 ETB	120	55.9
	1000–1500 ETB	39	12.2
	<1000 ETB	102	31.9

Have mass media	Yes	254	79.4
	No	66	20.6

ETB = Ethiopian Birr.

**Table 2:** Obstetrics complications and reproductive characteristics of the study participants in Hawassa Zuria district, Sidama Regional State Ethiopia, 2020.

Variables	Categories	Frequency	Percentage
Parity	<4	194	60.6
	≥4	126	39.4
Condition of pregnancy	Planned and supported	147	45.9
	Unplanned but supported	83	26.0
	Unplanned and unsupported	90	28.1
Had ANC visit	Yes	261	81.6
	No	59	18.4
Had complication during pregnancy	Yes	68	21.3
	No	252	78.8
Had complication during delivery	Yes	65	20.3
	No	255	79.7
Had complication after delivery	Yes	76	23.8
	No	244	76.2
Mode of delivery for the last birth	Normal	240	75.0
	Instrumental	54	16.9
	Surgery	26	8.1

**Table 3:** Awareness and attitude of the study participants on EPNC services and danger signs after birth in Hawassa Zuria district, Sidama Regional State, Ethiopia, 2020.

Variables	Categories	Frequencies	Percent
Had awareness on EPNC	Yes	176	55.0
	No	144	45.0
Had awareness on postnatal danger sign	Yes	135	42.2
	No	185	57.8
Common postnatal danger signs mentioned	Vaginal bleeding	85	26.7
	Severe abdominal pain	85	26.7
	Headache	55	17.0
	High-grade fever	50	15.6
	Blurring of vision	45	14.0
Attitude towards EPNC	Positive	175	54.7
	Negative	145	45.3

**Table 4:** Health-care provider and facility-related factors affecting utilization of EPNC in Hawassa Zuria district, Sidama Regional State, Ethiopia 2020.

Variables	Categories	Frequencies	Percent
Time taken to reach health facility	<30 minutes	179	55.9
	30 min–1 hour	118	36.9
	>1 hour	23	7.2
Place of delivery	Health facility	285	89.1
	Home	35	10.9
Appointed by health professionals for EPNC (*n* = 285)	Yes	136	47.7
	No	149	52.3
Time of stay at health facility after delivery (*n* = 285)	≥24 hours	138	48.4
	<24 hours	147	51.6
Reasons for not using EPNC services	Lack of information	90	40.0
	Lack of time	70	31.0
	Unwanted pregnancy	35	15.6
	Far health facility	30	13.4

**Table 5:** Factors associated with EPNC service utilization in Hawassa Zuria district, Sidama Regional State, Ethiopia, 2020.

Variables and categories	Utilized EPNC		COR (95%CI)	AOR (95% CI)
	Yes	No		
Age of the respondents				
<25	52	73	3.45 (1.72, 6.91)	**3.22 (1.37, 7.53)** ^*∗*^
25–35	30	89	1.63 (0.79, 3.37)	1.64 (0.73, 3.63)
>35	13	63	1	1

Educational status of the mother				
Secondary school and above	41	59	2.73 (1.34, 5.54)	1.23 (0.56, 2.68)
Primary school	40	111	1.41 (0.71, 2.82)	1.76 (0.78, 3.93)
No normal education	14	59	1	1

Parity				
<4	66	128	1.72 (1.03, 2.87)	1.45 (0.73, 2.88)
≥4	29	97	1	1

Condition of the last pregnancy				
Planned and supported	58	89	2.60 (1.41, 4.81)	**2.28 (1.15, 4.52)** ^*∗*^
Unplanned but supported	19	64	1.18 (0.57, 2.45)	1.11 (0.48, 2.57)
Unplanned and unsupported	18	72	1	1

Had ANC visit				
Yes	80	181	1.29 (0.68, 2.46)	0.75 (0.35, 1.60)
No	15	44	1	1

Faced complication during last pregnancy				
Yes	26	42	1.64 (0.93, 2.88)	1.42 (0.72, 2.79)
No	69	183	1	1

Faced complication after delivery				
Yes	30	46	1.79 (1.04, 3.08)	1.88 (0.99, 3.58)
No	65	179	1	1

Had awareness on obstetric danger sign and symptoms				
Yes	55	80	2.49 (1.52, 4.069)	**2.21 (1.27, 3.87)** ^*∗*^
No	40	145	1	1

Attitude towards EPNC				
Positive	71	104	3.44 (2.022, 5.858)	**3.50 (1.95, 6.33)** ^*∗∗*^
Negative	24	121	1	1

^*∗*^Statistically significant at *P* < 0.05, ^*∗∗*^statistically significant at *P* < 0.001.

## Data Availability

The finding of this study is generated from the data collected and analyzed based on stated methods and materials. The original data supporting these findings are available from the corresponding author on reasonable request.

## Abbreviations

ANC: Antenatal care
AOR: Adjusted odds ratio
COR: Crude odds ratio
EPNC: Early postnatal care
PNC: Postnatal care
SPSS: Statistical package for social science
WHO: World Health Organization.
